# Novel Carbon Fibre Composite Centrifugal Impeller Design, Numerical Analysis, Manufacturing and Experimental Evaluations

**DOI:** 10.3390/polym13193432

**Published:** 2021-10-07

**Authors:** Radu Mihalache, Ionut Sebastian Vintila, Marius Deaconu, Mihail Sima, Ion Malael, Alexandru Tudorache, Dragos Mihai

**Affiliations:** Romanian Research and Development Institute for Gas Turbines COMOTI, 061126 Bucharest, Romania; marius.deaconu@comoti.ro (M.D.); mihail.sima@comoti.ro (M.S.); ion.malael@comoti.ro (I.M.); alexandru.tudorache@comoti.ro (A.T.); dragos.mihai@comoti.ro (D.M.)

**Keywords:** composite materials, composite impeller, centrifugal compressor, composite impeller balancing

## Abstract

This paper presents an experimental investigation on using high strength-to-weight composite materials to reduce the mass of a centrifugal compressor impeller by 600%. By reducing the blades number from 17 to 7 and by doubling their thickness, the compression ratio and efficiency were maintained close to the reference metallic impeller. Using autoclave technology, seven composite blades were manufactured individually and assembled to form the impeller. After manufacturing, small deviations were found at the blade’s tip. As these deviations were found to be symmetrical, impeller balancing was successfully performed removing a total of 45 g of mass, followed by an experimental test on a dedicated test bench. Experimental testing identified the resonant frequencies of the composite centrifugal impeller at 13.43 Hz 805 rot/min and at 77 Hz with a 0.1 mm/s amplitude at 4400 rot/min, highlighting feasibility and the advantage of a composite compressor impeller design with application in centrifugal compressors and rotating machine assemblies and sub-assemblies. As there are numerous numerical investigations performed on the strength analysis and on the lay-up orientations mechanical behaviour for polymer composite materials with respect to the design of centrifugal impellers, no experimental evaluations in relevant working conditions have been performed to date. As the paper contains relevant experimental data on the subject, the outcome of the paper may aid the oil and gas or aviation industries.

## 1. Introduction

The current tendency for energy, aviation and many other industrial fields is to reduce fuel consumption and polluting emissions, represented by carbon dioxide (CO_2_) and nitrogen oxides (NO_x_). Through this, the aim is to reduce the environmental impact of these pollutants and their effects on climate change. Such pollutant reductions can be achieved by weight reduction in overall assemblies with focus on rotary sub-assemblies in industrial machines and in gas turbines. As carbon fibre composite materials offer a well-known low-mass high-strength ratio and are used considerably in the aviation industry, these materials have also gained interest in the oil and gas industry for manufacturing of risers, drill pipes and tubing, pressure vessels, tanks and pipe systems for fluid transport [1], but not yet in the rotary assemblies or sub-assemblies. Mallick [2] presented the use of thermoplastic and thermoset composites as an appropriate and affordable response to the manufacturing methods for high performance rotating machine components as motors and high-speed impellers. Depending on the application, up to 20% energy savings can be achieved by using composite impellers [3,4], as their lower weight reduces the start-up loads and shaft deflections, also allowing the composite rotating part to run with tighter clearance thus increasing the efficiency.

For the aerospace industry, there is an increasing demand for composite materials in rotary components, aiming to reduce the mass and noise in jet engines. In this regard, several studies have been conducted towards the design and testing of composite axial compressor blades with major focus on reducing vibration behaviour. Most of the studies in composite impellers, rotors, impeller/rotor blades or other composite rotary components have been carried out by numerical modelling, but only a few have been evaluated experimentally. An evaluation of first damage modes has been performed by Pollayi and Yu [5] with respect to matrix micro-cracking and cross-sectional stiffness for composite rotor blades using vibrational-asymptotical beam sectional (VABS) and geometrically exact beam theory (GEBT) analyses. The matrix cracking saturation density was found to be the starting point for the damage modes, delamination or debonding. Stevens [6] and Pawar [7,8] performed numerical analyses of a composite helicopter rotor to evaluate the effect of matrix cracking and first failure mode in the composite part, concluding that the stiffness degradation of the composite is correlated to the life consumption of the rotor blade. As the appearance of cracks in composite blades lead to degradation in flapwise and lagwise performances of rotor blades, a damage detection strategy was proposed by Lakshmanan and Pines [9], considering the use of structural waves to detect these kinds of damages. Experimental validations demonstrated the success of the model in detecting chordwise cracks using a least square optimisation and scattering coefficient, with an error of 20% for depth and 7% for location. Mao and Mahadevan [10] developed a mathematical model for fatigue damage evolution in composite materials. The results showed that there are three damage evolution stages of fatigue loading, a fast increase in fatigue damage due to the occurrence of multiple damage modes within the material, a steady and slow increase and again a fast damage growth due to fibre fracture. An approach on modelling composite beam structures has been addressed by Hodges and Yu [11], for application in wind turbine, helicopter blades and other rotary assemblies. The methodology accounts for modifying the 3D model into a 1D beam model and a 2D cross-sectional model, thus leading to significant reduction in computational effort. Results obtained demonstrate the considered methodology. A brief strength analysis was made by Lin et al. [12], for a carbon fibre reinforced polymer (CFRP) composite impeller for centrifugal pumps, having short and long blades, indicating that the at a speed of 3600 rot/min, the highest stress of 23.6 MPa is located at the blade pressure side, near the blade root, meeting the requirements of the centrifugal pump. It was concluded that the strength of the carbon fibre impeller can meet the requirement of the centrifugal pumps where the largest stress was found around the blades root on the blades pressure side, thus reducing the fatigue limit. Hong et al. [13] studied the design of a composite propeller blade with a focus on damping behaviour, by performing a series of modal and structural analyses and varying the material layup. It was observed that the 0° and 45° fibre orientation influence greatly the static damping capacity of the composite blade. However, the dynamic response could be improved by means of design optimisation. Vipin and Jiju [14] numerically investigated and compared the tangential and axial forces deformation and thermal behaviour of a steam turbine blade made of 304 stainless steel, aramid, glass and carbon fibres composites. The authors concluded that the carbon fibre material show more desirable mechanical characteristics compared to the other materials.

Wollmann et al. [15] evaluated the vibration amplitudes and damping behaviour for composite compressor blades. By applying a force equal to the rotational speed of 16,000 rot/min, a 10.5% maximum relative deviation was achieved between the numerical and experimental Eigenmodes, for a ±45° layer orientation. Gude et al. [16] studied the assessment of a simulation model for the radial expansion and vibration response up to a speed of 12,000 rot/min, for a composite rotor blade manufactured out of glass fibre reinforced polymer (GFRP) composites, with a layup of 20 bidirectional layers [45°/0°/−45°/90°/45°/0°/−45°/90°/45°/0°]. It was concluded that the rotor had a non-linear behaviour, validated by the numerical method and a very good correlation of vibration stress states between simulation and experimental measurements has been identified. Martynyuk et al. [17,18] have presented the possibility of using polymer composites in highly loaded propulsion systems correlated with the analysis of the centrifugal compressor impeller thermal state, illustrating that a composite centrifugal impeller could be manufactured using a heat-resistant thermosetting binder that increases the operating temperatures up to 350 °C, offering a 45% mass reduction of the impeller. A preliminary investigation of using CFRP composites in the fabrication of composite impeller blades was performed by [19], evaluating technological aspects and dimension stability of the composite blades after processing, by using 3D printed thermoplastics moulds. Although good dimensional stability was obtained, higher dimensional accuracies and mechanical properties can be obtained by applying pressure and elevated temperature that are not suitable for printed thermoplastic materials.

A study on design, analysis and optimisation was performed by Dhere et al. [20] for a composite submersible pump impeller. A weight reduction of 76% was obtained as a comparison to the reference metallic impeller. The glass fibre pump impeller was tested under compression loads to evaluate the deformations on the glass fibre impeller and to compare it with the finite element analysis (FEA) results, showing a convergence between the two. A failure case report of a fibre reinforced impeller was released by UTComp [21] reporting that the failure occurred during commissioning activities, at 1252 rot/min, after 200 h of operation. The impeller blade including a portion of the rim received for analysis was cracked on the pressure side and leading edge. In addition, the backplate of the rim presented cracks in the connection with the blade. The identified cracks were limited to the resin and did not rupture the fibres. It was concluded that bending moments during cyclic loading was the cause of failure. A fibre glass centrifugal pump impeller was designed by Kumar et al. [22], subjected to static and dynamic numerical analyses and the results were compared to various metallic alloys. It was concluded that the fibre glass/epoxy composite present better stress bearing capacity compared to other metallic materials, except titanium alloys. The production method and use of elastomeric matrix composites has been investigated by Chukov et al. [23] that could be used to manufacture products with complex shapes as pump impellers. Two impeller prototypes were designed, manufactured and analysed. It was reported that good adhesion between fillers and carbonized matrix was obtained, showing good mechanical properties, as closed to those of carbon-carbon composites. A parametric modelling method of composite blade with typical profile and high simulation degree for designing a composite rotor blade was assessed by Ma et al. [24]. The numerical investigations show that the proposed model can effectively analyse the aeroelastic characteristics of general composite rotors.

The current study proposes an optimised centrifugal impeller design, with a total mass reduction of about 600% as compared to the metallic (17−4 PH) reference one. By computing aerodynamic and structural analyses, the final thickness of the composite blades and total number of layers has been found. Taking in consideration the autoclave technology, 7 carbon fibre composite blades have been manufactured and assembled to form the full composite centrifugal impeller. The composite impeller was then subjected to balancing operations specific to rotary machines and mounted on a test rig for experimental evaluations. When compared to the existing bibliographic study, a major novelty aspect of the present paper is represented by the full fabrication process and technology used for the composite centrifugal impeller as well as the balancing and experimental investigations performed by the authors. The results showed that the optimised composite centrifugal impeller design could be used in a high-speed rotary assembly (as oil and gas industry) as the vibration levels are within imposed limits.

Considering the preliminary work mentioned above and the work performed by the authors to assess the absence of such high-speed rotary composite parts (no centrifugal composite impellers are presented to be manufactured or tested up to date), the outcome of this study may benefit the oil and gas industries and also the aviation industry that make use of such high-speed rotary assemblies, by allowing the composite rotating part to run with tighter clearance (due its low mass) thus increasing the efficiency. Future aspects envisaged by the authors are the one-shot fabrication process for such a composite impeller and the evaluation of hydraulic performances (pressure and flow rate).

## 2. Materials and Methods

**Figure 1:**
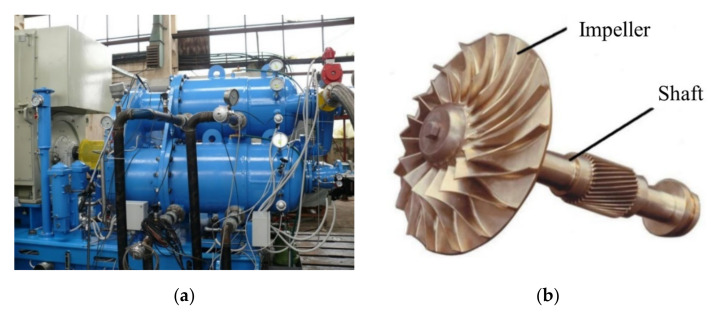
Representative images of (**a**) COMOTI CCAE 9-125 centrifugal compressor and (**b**) reference metallic impeller taken as input.

The reference centrifugal impeller (shown in Figure 1b) has a weight of 22 kg, is made of 17-4 PH stainless steel with 17 blades, Ø372.5 mm × 78 mm height, operates at a speed of 17,050 rot/min, providing a flow rate of 4.25 kg/s, designed for stage 1 of the CCAE 9-125 centrifugal compressor (Figure 1a) used in the gas/oil industry (discharge pressure 8.7 bar, flow 5200 Nm^3^/h, power 510 kW), having three compression stages and intermediate cooling, owned by the Romanian Research and Development Institute for Gas Turbines COMOTI [25].

### 2.1. Materials

A CFRP prepreg (Hexcel Corporation) having an epoxydic matrix (M49) with a high strength bi-axial carbon fibre weave (T300) was used for the manufacturing of individual composite blades and for the final composite impeller. A 2024 Aluminium alloy was used only for manufacturing the blade moulds and a support component aiding the final integration and assembly of the composite impeller. Materials properties are presented in Table 1, as indicated by their data sheets. As the aluminium alloy was used only for mould manufacturing, only its physical properties are presented in Table 1.

### 2.2. Numerical Analysis of the New Impeller Design

Preliminary numerical analyses were made using Ansys CFX software (V19.2) to define the new impeller design with respect to the thickness and number of blades, while keeping the same blade profile and mechanical interface.

#### 2.2.1. Composite Impeller Aerodynamic Analysis

**Figure 2:**
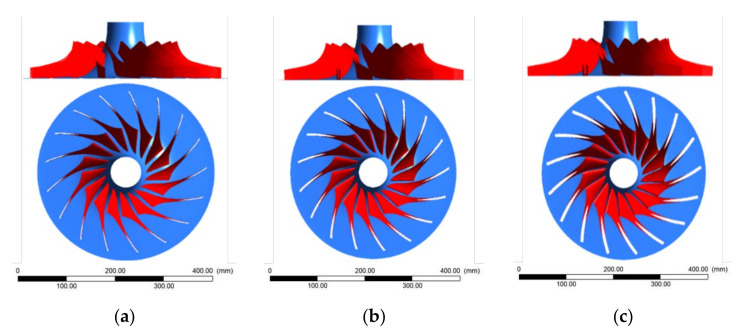
Modelling representation of the thickened blades shape and their intersection with the peak flow channel curve for the 17 blades impeller: (**a**) blade model 1×; (**b**) blade model 2×; (**c**) blade model 3×.

**Figure 3:**
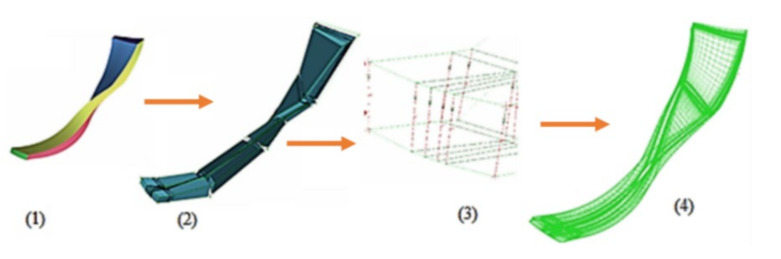
Topological isomorphism for the pre-mesh and domain discretization, where **(1**) analysis domain; (**2**) blocking structure; (**3**) edge parameters for the blocking structure; (**4**) discretization grid.

Following the reference metallic 17-4PH impeller geometry, the thickness of the blades was modified up to three times as compared to the reference model (shown in Figure 2), in order to analyse the aerodynamic characteristics of the new model. The number of blades was also influenced by the blades thickening, varying from 17 (as the metallic reference), to 15, 13 and 7 blades, as seen in Table 2. Figure 3 presents the discretization grid for one of the three geometries shown in Figure 2, with 539,248 elements, considering air as working fluid at a reference pressure of 0 bar. The boundary conditions set for the analysis were a pressure of 101,353 Pa and a temperature of 288 K at the inlet and a 4.25 kg/s flow rate at the outlet, with a no-slip condition for the blade walls. The discretization was based on a topological isomorphism (blocking structure), having the first cell height corresponding to a value of the dimensionless thickness of y+ = ~1 [28,29]. For the current case study, it was considered that the y+ value should be as low as possible. As the flow for this impeller geometry is periodic, the analysis was performed only for a sector, as presented in Figure 3.

#### 2.2.2. Composite Impeller Structural Analysis

**Figure 4:**
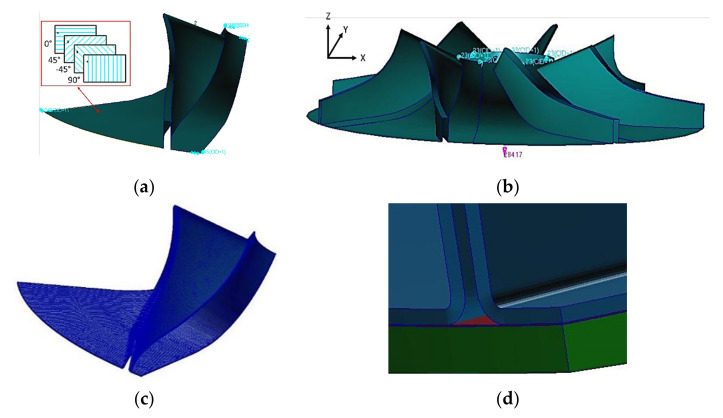
Boundary conditions for 2D shell element of the (**a**) blade sector with composite lay-up and (**b**) impeller blading; and for 3D element of (**c**) disc and blading element and (**d**) interface location in assembly.

The structures of the laminate composite blade and of the rotor (blade and disc) were modelled using two approaches, namely a 2D finite elements model (FEM) and 3D finite elements model. Tsai-Wu and Chang criteria were used for the structural analysis, performed with 2D type shell elements to define the required numbers of composite layers for the blades and impeller disc in order to respect its functional requirements. Thus, the blading was modelled using 4 node shell elements and the composite layers were defined by the composite material properties, presented in Table 1. Boundary conditions for the flow channel surface geometry are presented in Figure 4a,b and consisted in fixing the impeller in plane Z = 0 and axial symmetry on the circular contour near the impeller inlet. The state of deformation of a ply was calculated using the deformation of the reference surface and offset distance between ply and reference surface. Using the deformation state, the stress tensor was calculated using material constitutive law. The 2D FEM analysis was performed by varying the number of layers on the blades as well as on the disc to achieve a final construction design. The fibre reinforced material of a ply was Also modelled using a 2D orthotropic elastic material with equivalent mechanical properties calculated using homogenisation rules. The main limitation of the 2D finite elements model is the way of strain and stress states that are calculated [30].

The stress on the normal direction to reference surface was underestimated on the corner joint or T-joint. In order to avoid this under estimation, the, Hashin-Fabric criterion was used for the analysis performed with 3D type elements. The blading was modelled with CHEXA elements (parallelepipeds) and the composite material was modelled with PCOMPLS using MSC/NASTRAN software, the disc was modelled with orthotropic materials and the interfaces with CHEXA elements and isotropic materials, presented in Figure 4c,d. Thus, at the corner joint and T-joint the tensile force acting on one corner surface is transferred as normal force on the other corner surface. In the 2D and 3D finite elements model of the composite laminate (reinforced with 2 directional fibres), the state of stress doesn’t take in account the influence of the micro-cracks on the fracture criteria (delamination between fibre and matrix, cracks on the matrix/fibre, etc.) [31]. The applied load consisted in the action of centripetal forces caused by the reaction to the rotational motion at 17,250 rot/min.

### 2.3. Balancing Procedure

**Figure 5:**
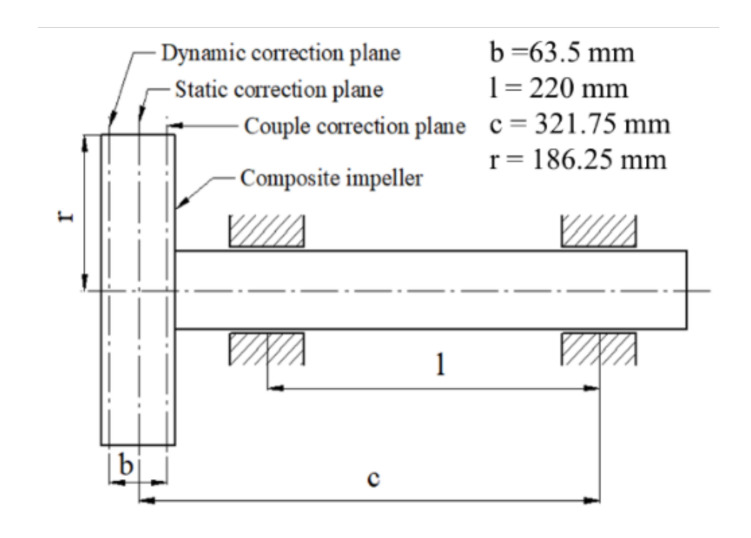
Schematic representation of the balancing configuration.

A preliminary study for the balancing procedure for a composite centrifugal compressor impeller was assessed by Popescu [32], where it was found that a full composite impeller led to the impossibility of correction by material removal. Furthermore, the low weight of this type of impeller leads to a much smaller values of permissible residual unbalance, requiring a more accurate dynamic balancing. Thus, the addition of 304 stainless steel rings on the back of the composite impeller could ensure the dynamic balancing. A theoretical calculation of the maximum accepted unbalance and balancing parameters (Figure 5) was based on ISO 1940-1:2003 standard [33]. The theoretical calculations were made using Equations (A1) to (A8) presented in Appendix A. The theoretical mass that shall be removed during balancing operation was found at 52.44 g.

### 2.4. Experimental Test Bench

**Figure 6:**
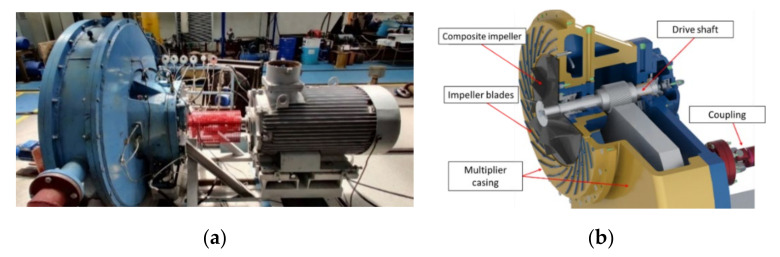
Experimental (**a**) compressor test bench and (**b**) schematic illustration of rotary assembly.

For validating the balancing precision and evaluating the mechanical performances of the composite centrifugal impeller, a compressor test bench, illustrated in Figure 6, was used. The test bench provided a 200 W nominal power, a maximum speed of 3000 rot/min and a compression equipment unit with integrated multiplier with a ratio of 5.78:1.

Vibration levels were measured during testing by means of two PCB 032C03 accelerometers (PCB Piezotronics, New York, NY, USA) placed at the multiplier inlet and outlet, a Brüel and Kjær 4294 calibration unit (Brüel & Kjær, Skodsborgvej, Denmark) and a DeweSoft Sirius (DEWESoft d.o.o., Trbovlje, Slovenia) data acquisition and analysis system. The nominal vibration level was set at 2.3 mm/s, the vibration warning level at 4.5 mm/s and the maximum allowed vibration level at 7.1 mm/s, as indicated by ISO 2372-1974 [34].

## 3. Results and Discussions

### 3.1. Aerodynamic Analysis

**Figure 7:**
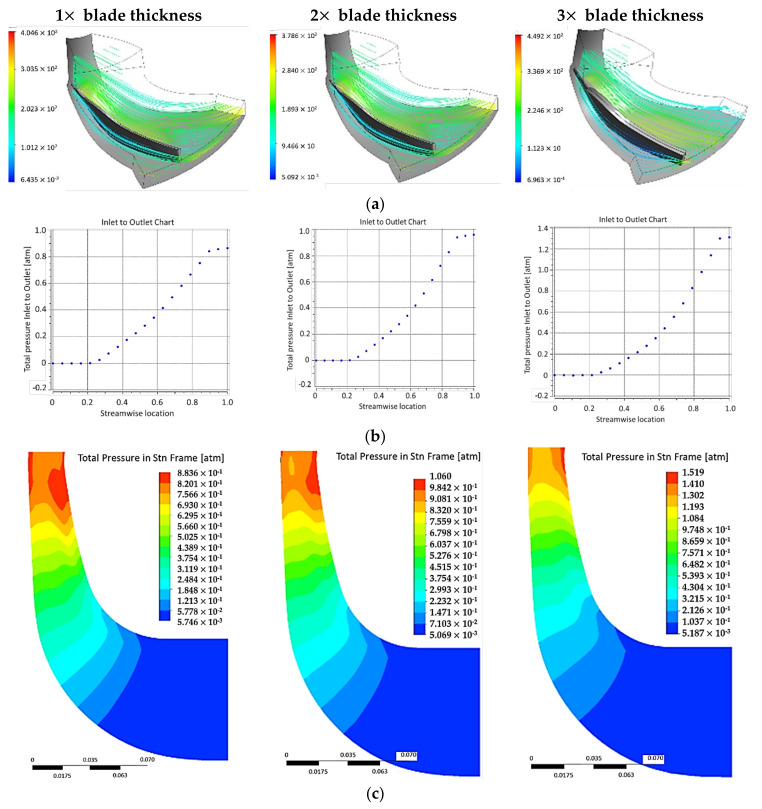
Analyses results presenting (**a**) stream lines; (**b**) pressure variation from inlet to outlet and (**c**) pressure variation in meridional plane, for the impeller case with 7 blades.

With respect to the boundary conditions previously mentioned, 12 analyses were performed and presented in Table 3, considering the variation of blades number and thickness and their impact on the impeller performance. It was observed that by thickening the blades in the case of an impeller with a reduced blade number, lead to better performances of the impeller as compared to the same thickening for a larger number of blades, as seen in Table 3. The values reported with “N/A“ indicate that the corresponding geometry is not applicable for the impeller working conditions. Bolded values represent the performance values for the reference metallic impeller at nominal (1×—3 mm) thickness. The stream lines and pressure variation results for the impeller with 7 blades are presented in Figure 7. For the 7 blades impeller case, no boundary layer separation was observed for the blade walls and also no recirculation area was present that could lead to significant pressure losses. By using a thicker blade, a 15% pressure increase was found for the outlet, which also influences the impeller efficiency, reaching 91.5% (Table 3).

Of the total impeller cases calculated and presented in Table 3, the centrifugal impeller having 7 blades with a thickness of 2x (6 mm) was selected as indicated in Table 4, following a score methodology using equation (9) presented in Appendix A, with respect to the reference impeller compression ratio (2.067), power (310.4 kW) and efficiency (91.8%). It is to be mentioned that the 6 mm blade thickness consisted in a 1.5 mm blade wall and a 3 mm gap left for autoclave manufacturing technology (vacuum bag to be inserted).

### 3.2. Structural Analysis

**Figure 8:**
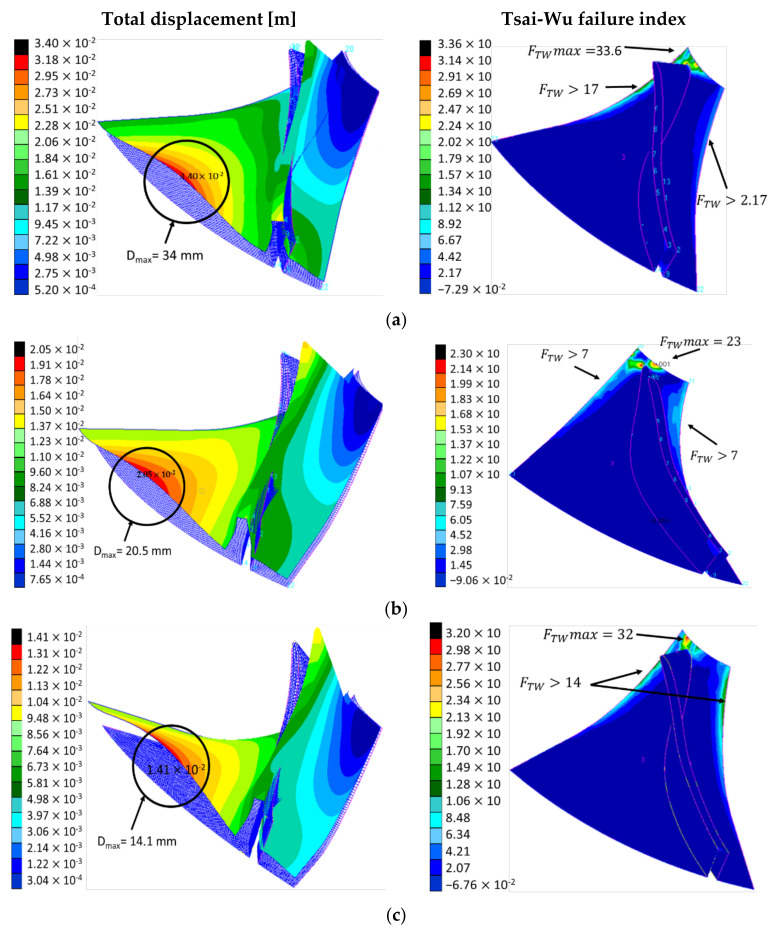
Distribution for the total displacement and Tsai-Wu failure index for the blade modelled with (**a**) 4 composite layers; (**b**) 6 composite layers; (**c**) 8 composite layers.

The total displacements and failure index for the blades consisting in 4, 6 and 8 composite layers is presented in Figure 8. The blade with only 4 composite layers has the highest deformation with 34 mm, and fails at the hub interface, having a failure index of 33.6 (failure occurs at FI > 1). It was also found that the failure index on the blade side is higher than the failure limit. By doubling the composite layers, the total deformation is reduced by more than double and the failure index is also reduced. However, not all three configurations withstand the centrifugal loads corresponding to the rotor assembly speed of 17,050 rot/min.

**Figure 9:**
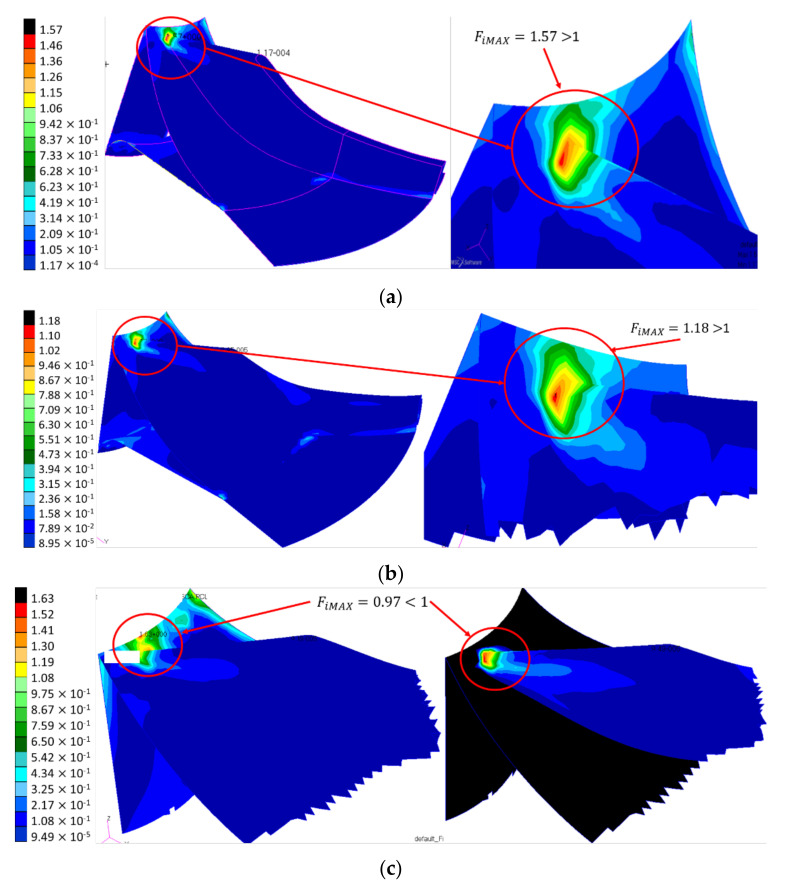
Chang failure index for the different number of composite layers on the disc and blade: (**a**) Disc 22 layers; Blade 7 layers; (**b**) Disc 22 layers; Blade 7 layers bottom and 5 layers top; (**c**) Disc 44 layers; Blade 7 layers bottom and 5 layers top.

To further consolidate and optimize the impeller geometry the impeller disc was modelled with median surfaces and shell elements using Chang criterion, assuring the axial symmetry by alternating the composite layers from 5° to 5° orientation. In this regard, three analyses were performed, by modelling a combination of 22 composite layers for the disc and 7 composite layers for the blades; a combination of 22 composite layers for the disc, 7 composite layers for blades bottom and 5 layers for the blade top; a combination of 44 composite layers for the disc, 7 composite layers for blades bottom and 5 layers for the blade top. The results for the Chang failure index are shown in Figure 9.

According to Chang criterion results, the failure occurs due to flexural loads caused by the difference between the composite layer numbers and a uniform distribution of mass on the blade, increasing the inertial forces and stresses. Reducing the mass from the blade’s top has reduced the failure index distribution by 25%, from 1.57 to 1.18, but still not compliant, as it should be below 1. By doubling the composite layers on the disc and maintaining the same lay-up configuration for the blade, the blade failure index was reduced to 0.97. However, a maximum failure index of 1.68 was found at the hub–flow channel interface, appearing as a crack-like phenomena due to the thickness difference between the two structures, as also identified by [5]. Thus, a strength analysis was performed on an impeller sector using 3D shell elements, taking into consideration the boundary conditions from Figure 4b and the composite material lay-up having the exterior layer (layer 10001) at +45° and the following layers at [0°/±45°]_6_.

**Figure 10:**
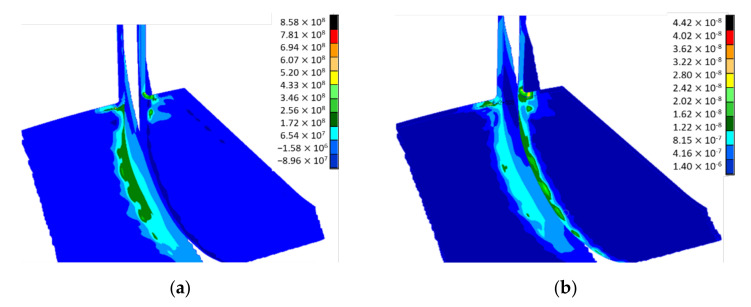
Maximum (**a**) principal stress and (**b**) shear stress for layer 10001.

**Figure 11:**
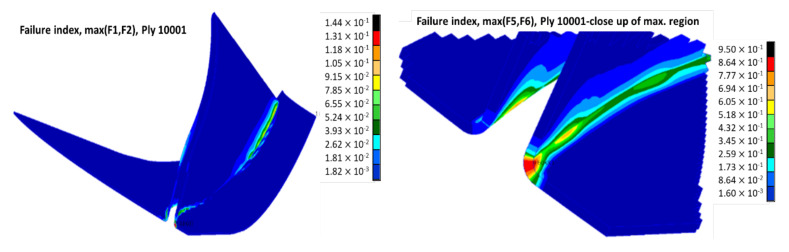
Maximum Hashin-Fabric failure index distribution values for layer 10001.

**Figure 12:**
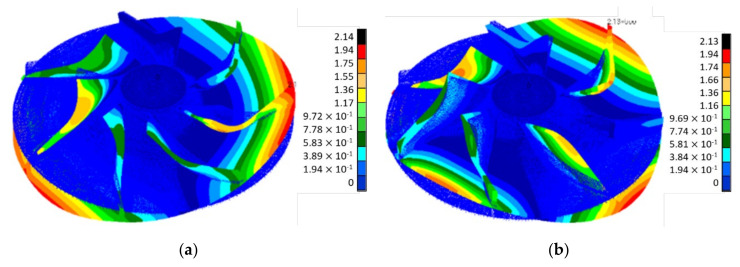
Results for the (**a**) first and (**b**) second Eigenmode of the composite impeller.

The maximum principal stress was found at 868 MPa located on layer 10001 for the disc-blade interface, near the impeller outlet, but within the composite material ultimate strength limits (Table 1), also the maximum shear stress was found at 442 MPa on the same region (Figure 10). Maximum values appear due to disc stiffness at the disc-blade interface, which creates a stress concentration phenomenon as well as a flexural phenomenon under the action of the centrifugal forces. The flexural phenomena act as a tensile stress in the composite layer that assures the bonding between the disc and the blading. For this layer, Hasin-Fabric criteria was applied to evaluate the failure index distribution, and the results are presented in Figure 11. The maximum value for the failure index was found at 0.95 only on layer 10001 on the blade and disc joining edge. The small 5% difference until the failure limit of 1 implies a verification of the corresponding edge on the composite impeller after experimental testing, identifying any potential micro-cracks, delamination, etc.). It was observed that the main Eigenmodes are characterized by a bending mode of the disc having a constant participation of the blades as well. The Eigenvectors of the first two mode shapes are presented in Figure 12.

### 3.3. Blades and Impeller Manufacturing

**Figure 13:**
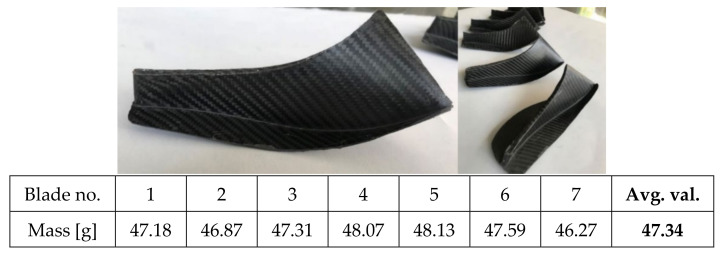
Composite impeller blades after manufacturing process.

Using the corresponding pressure side and suction side Aluminium moulds, 7 composite impeller blades have been manufactured using autoclave technology and presented in Figure 13. The manufacturing parameters used were a temperature of 120 °C with a heating rate of 3 ± 0.1 °C/min, a pressure of 7 bars in the autoclave, a vacuum of 0.9 mbar (vacuum bag) for 120 min with a cooling rate of 4 ± 0.5 °C/min until 40 °C. The blading was trimmed to avoid mass differences between blades, roughness measurements were also performed on the blading area of each composite blade, using a Mahr Surf PS10 instrument (Mahr GmbH, Göttingen, Germany), over a distance of 10 mm with a 1 mm/s rate. Due to the curvature of the blades, roughness was not measured on either suction side or pressure of the composite blade but were considered to be within the obtained values identified for the blading. The average results are presented in Table 5, as an average of three measurements, indicating that the obtained roughness is between the imposed technological values of Ra 0.8–1.6. This verifies also the manufacturing process using the autoclave technology. In addition, the Ra is considered to be a critical point in the hydraulic performances of such a component, as it can influence the flow, thus affecting the overall hydraulic performance.

**Figure 14:**
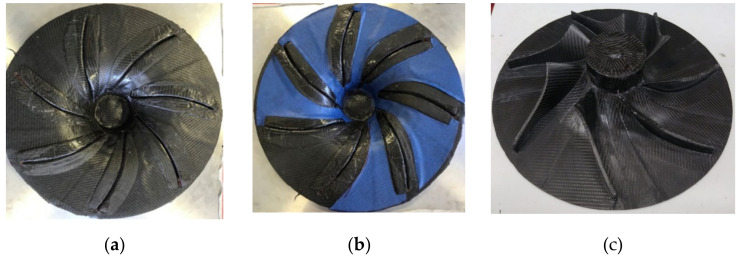
Representations of the composite impeller first manufacturing step: (**a**) positioning of the 7 composite blades on the base composite layers; (**b**) composite layers applied to uniform the thickness; (**c**) composite impeller after first manufacturing process.

**Figure 15:**
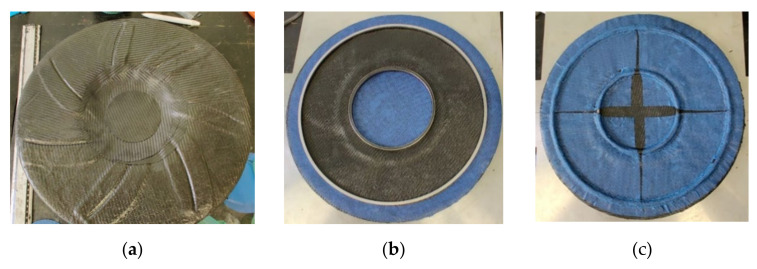
Representations of the composite impeller second manufacturing step: (**a**) composite layers to form the back of the impeller; (**b**) metallic rings for balancing operations; (**c**) composite layers to fix the metallic rings.

The composite impeller manufacturing process was performed in two steps. Firstly, the integration of each individual blade by applying a base of three composite layers on the Aluminium support component mould (Figure 14a), followed by positioning the blades and applying another 15 composite layers between the blades to reduce the height difference, as seen in Figure 14b. Further, 20 composite layers (Ø85 mm) were placed in the hub area to preliminary form the hub-shaft interface. The assembly was vacuumed and cured using the same parameters as for the blades manufacturing process. The impeller blading is presented in Figure 14c. The second manufacturing step consisted in applying a number of 400 composite layers (Ø85 mm) to form the impeller hub. After each 20 composite layers, a set of 10 composite stripes (15 mm × 150 mm) were placed to create an interface with the disc, to prevent delamination during mechanical processing. Following, a number of 22 composite layers of Ø110 mm and 22 layers of Ø400 mm were placed to form the disc, obtaining a total of 44 layers for the disc, respecting the layers’ number defined through FEA. In addition, the two metallic rings were placed on the back of the impeller to aid the balancing procedure. The second manufacturing step is presented in Figure 15.

The obtained mass for the composite impeller after the first manufacturing step was 0.869 kg and 2.271 kg after the second step, reaching a 600% mass reduction compared to the metallic reference.

**Figure 16:**
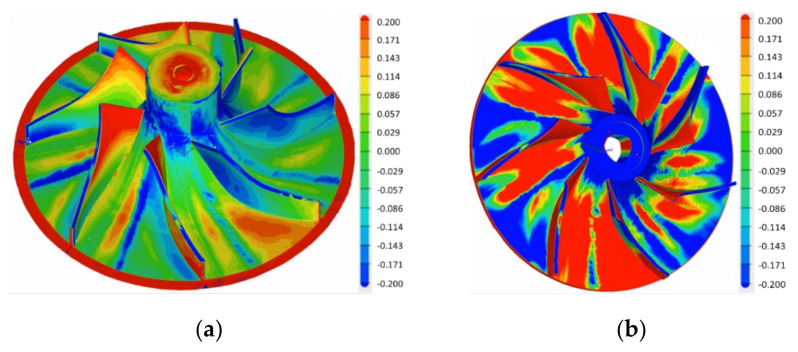
Composite impeller dimensional accuracy after (**a**) first and (**b**) second manufacturing phase.

**Figure 17:**
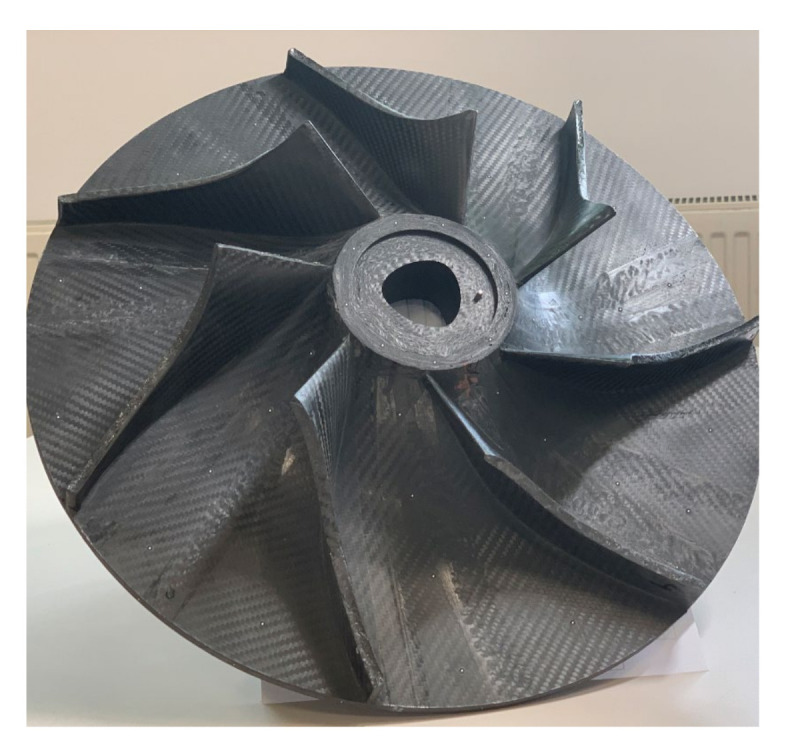
Composite centrifugal compressor impeller after machining.

Dimensional accuracy measurements were performed on the composite impeller, using ATOS Compact Scan 5M machine (GOM GmbH, Germany) integrated with GOM software with 2 × 5 × 106 pixels. After the first 3D dimensional measurement it was observed that impeller’s blading has a maximum deviation of ±0.186 mm (found at the blade tip) and is close to the imposed tolerances of ±0.15 mm. The red outer diameter area shown in Figure 16 represents an offset material, intentionally left to aid the removal of the impeller from the mould, that was machined after the second manufacturing step. For the second dimensional measurement, it was observed that the profile deviations are higher when compared to first analysis, mainly due to the absence of moulds in the second manufacturing process and due to the applied vacuum that induced small blade deviations, at their tip. However, the average maximum profile deviation was found at ±0.386 mm, located at the tip of the blades. As the deviations were found to be nearly symmetrical, it was considered that the composite impeller (shown in Figure 17) could undergo the balancing procedure and the experimental evaluation and verification on the dedicated test bench, aiming to validate the new design and the associated manufacturing technology.

**Figure 18:**
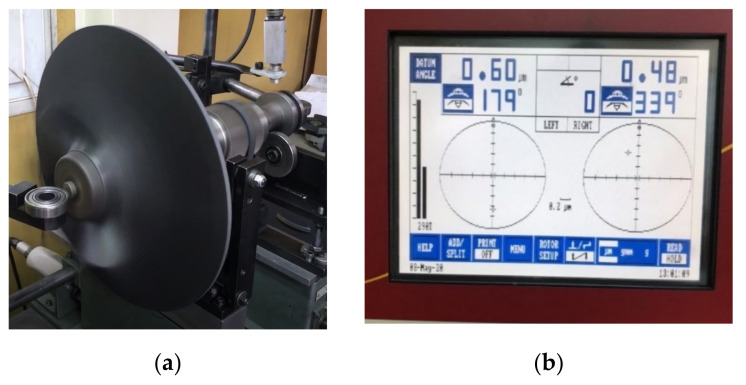
Representation of the (**a**) composite impeller balancing and (**b**) amplitude of unbalance identification.

**Figure 19:**
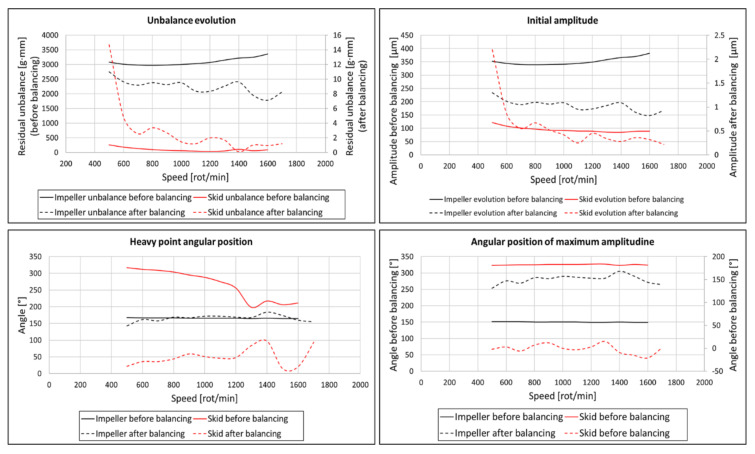
Composite impeller unbalance results before and after balancing operation.

The composite impeller balancing was performed on a B-Series IRD horizontal balancing machine (IRD^®^ LLC, Louisville, KT, USA), using a Model 290 IRD data acquisition aiming a balancing class of G2.5 and considering two correction planes. The driveshaft and the skid were previously balanced to reduce the possible unbalance. Composite impeller balancing procedure was performed in several iterations until the measured unbalanced was lower than the permissible one. It is to be noted that balancing was achieved having mass removal only from the outer metallic ring, highlighting the possibilities to further optimize the impeller design and mass by using only one balancing metallic ring. By slowly increasing the speed and analysing the unbalance, Figure 18, it can be concluded that the two-step manufacturing process for the composite impeller induces a significant residual unbalance, more exactly 3360 g∙mm at a speed of 1600 rot/min (Figure 19).

Following the balancing procedure, the residual unbalance and amplitude have decreased considerably, as seen in Figure 19. With respect to the heavy point angular position, it can be seen that the skid is still influenced by the impeller unbalance. However, these unbalance values are very low, namely 9.53 g∙mm (1.09 µm at 157°) in the left plane and 1.47 g∙mm (0.42 µm at 0°) in the right plane, being within the imposed limits of G2.5 class (17.3 g∙mm in the left plane and 3.24 g∙mm in the right plane). During the balancing procedure, a total of 45 g of metallic material was removed from the outer metallic ring. If the unbalance was higher, mass removal from the interior metallic ring would have been necessary.

An impact hammer test (Ping test) was also performed on the composite impeller and presented in extenso in [35] in order to determine the impeller and blades Eigenfrequencies. Values presented in Table 6 are shown as average values, by applying three independent shocks on each blade and on the impeller.

The comparative analysis of the Ping test results with that of the vibration calculation shows that the finite element model is adequate to the real one within the limits of the calculated deviations. The deviations related to the test results can be explained by the variation of the constructive parameters (uniform arrangement of the resin, homogeneous mechanical properties) as well as by the influence of the spectral components of the shock stress.

### 3.4. Test Bench Results

**Figure 20:**
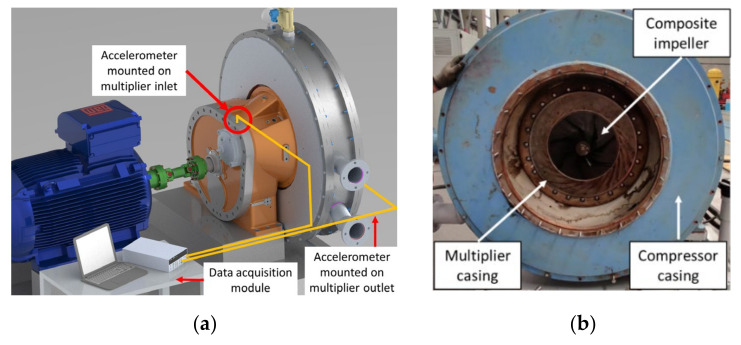
Composite impeller test set-up: (**a**) schematic representation of accelerometer positioning; (**b**) impeller position in the compressor assembly.

The composite impeller testing did not consider the compression degree or the discharged air flow due to the composite impeller dimensional deviations. Namely, blades tip non uniformity did not allow a full assembly of the test bench and the corresponding gap between the impeller and the stator casing was not in line with the testing constraints. Considering this, only vibration levels were measured to assess the design, manufacturing and balancing operations of the composite centrifugal compressor impeller and the integrity of such rotary component under high centrifugal forces, aiming to validate the numerical analysis. To measure the vibration level during testing, the two PCB 032C03 accelerometers were mounted near the multiplier inlet bearing and on the multiplier outlet, as seen in Figure 20a.

**Figure 21:**
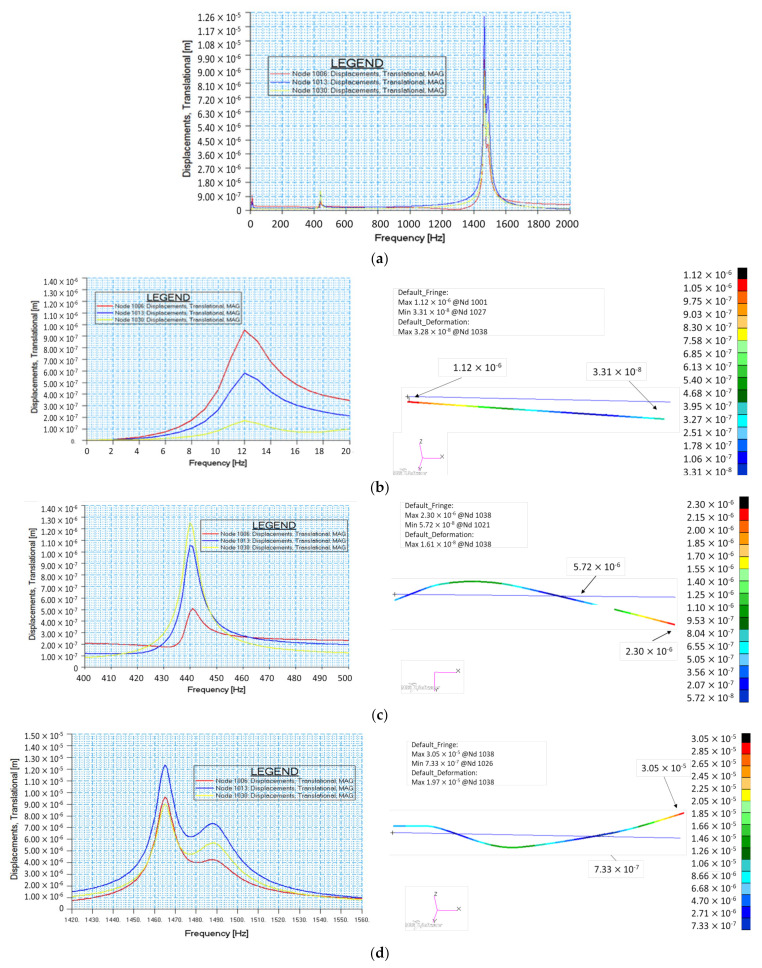
Critical frequency response analysis for (**a**) 0 to 2000 Hz; (**b**) F1 = 12 Hz and AF = 3.75; (**c**) F2 = 440 Hz and AF 73.33; (**d**) F3 = 1465 Hz and AF = 121.91.

The frequency response analysis of the impeller-shaft-bearing assembly was firstly numerically analysed as a function of centrifugal force of the impeller unbalance and validated by experimental testing on the test bench. Three critical frequencies and corresponding amplification factors (AF) were identified and presented in Figure 21. First critical frequency was identified at 12 Hz (960 rot/min), which corresponds to a conical Eigenvalue (rigid shaft); nevertheless, this does not affect the working regime of the impeller, as the speed is constantly increasing up to the nominal operating speed of 17,250 rot/min. As the operating speed was further increased, the second critical frequency was identified at 440 Hz (26,400 rot/min) that corresponds to a lateral bending phenomenon and this operating regime must be avoided for such composite rotary components. However, as previously mentioned, the nominal speed of impeller is 50% below the critical identified speed. The third critical speed was identified at 1465 Hz (87,900 rot/min) with a margin of safety bigger than 500% (F3/FN = 5.09) relative to nominal operational frequency FN at 287.5 Hz. Amplification factor was calculated in accordance with API 617 standard [36]. The first critical mode calculated with Frequency Response Analysis, F1 = 12 Hz is a conical mode shape, Figure 21e, where the deformed shape of the rotor assembly is a straight line (rotational speed applied to the deformed shape will generate a conical surface). It is to be noted that in Figure 21 Node 1006 is the composite rotor centre of mass, Node 1013 is the middle section of the bearing 1 and Node 1030 is the middle section of the bearing 2.

**Figure 22:**
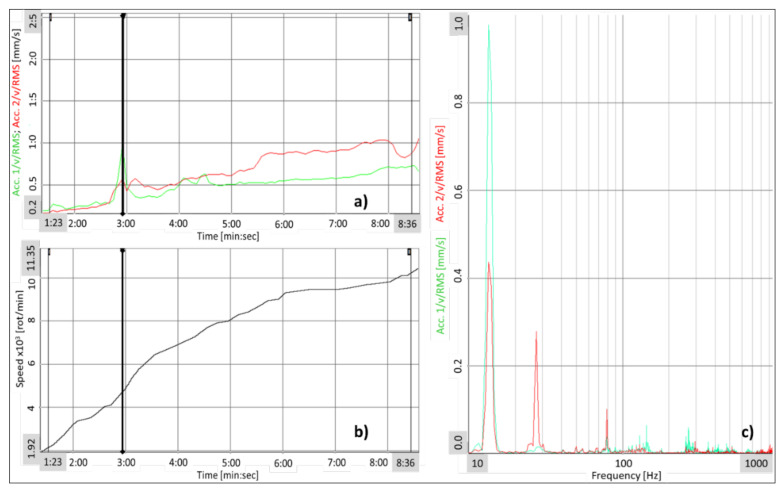
(**a**) Vibration level variation over time; (**b**) Speed variation over time; (**c**) Spectral analysis of speed (4400 rot/min) corresponding to cursor location from (**a**) and (**b**).

**Figure 23:**
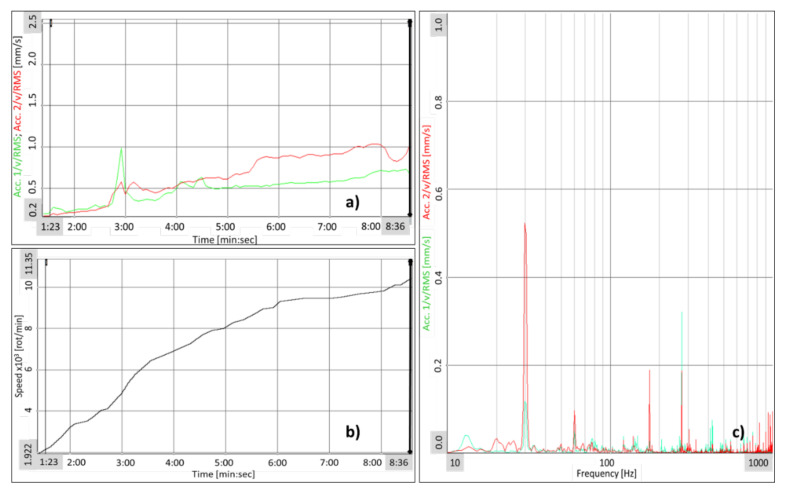
(**a**) Vibration level variation over time; (**b**) Speed variation over time; (**c**) Spectral analysis of speed (11,353 rot/min) corresponding to cursor location from (**a**) and (**b**).

A graphical representation of the root mean square (RMS) values for the compressor impeller speed is shown in Figure 22 as a function of time, correlated with spectral analysis. During the composite impeller testing, a rapid increase in vibration was identified at a speed of 4400 rot/min. Following a Fast Fourier Transformation (FFT) analysis, it was observed that the rapid increase in vibration was due to the spectral component measured at 13.43 Hz (805 rot/min) corresponding to first critical speed calculated with Frequency Response Analysis. Subsequently, the compressor speed was observed at 77 Hz frequency with a 0.1 mm/s amplitude, recorded by the accelerometer placed near the multiplier outlet bearing. At the speed of 8100 rot/min and 9200 rot/min, the FFT analysis showed that the measured vibrations are produced by the electric motor, the speed and amplitude being maintained at 0.1 mm/s. At the highest testing speed of 11,353 rot/min (Figure 23), the measured vibration level was constant, having the same previous values.

Vibration measurements for the composite centrifugal impeller during experimental testing showed that the rotary assembly had a constant vibration behaviour, indicating that the composite impeller balancing operation was performed successfully with no eccentricities on the composite impeller that could induce vibrations while operating as part of the compressor assembly.

Future works envisage a single-step manufacturing process for the composite centrifugal impeller and using the corresponding number of moulds to avoid profile deviations. This would allow to integrate the CFRP impeller on the experimental test bench in complete configuration in order to determine the hydraulic performances of the new impeller design.

## 4. Conclusions

A new design dedicated for autoclave technology (iteratively obtained by varying the number of blades and thickness) was proposed and experimentally evaluated for a CFRP composite centrifugal impeller. The implications of the manufacturing technology and balancing process over the vibration levels obtained during functional testing were investigated. Numerical analysis showed that by using a total of 7 blades with 6 mm thickness, the impeller theoretical efficiency has reached 98.15% when compared to the efficiency of the reference 17-4PH metallic impeller having 17 blades and 3 mm thickness. Moreover, using structural numerical analysis, a specific number of layers and orientation has been identified, namely 44 layers for the disc and 7 layers for the blades at a [0°/±45°]_6_ lay-up. The FEM analysis indicated a maximum value for the failure index of 0.95 and a maximum principal stress of 868 MPa located on the outer layer, lower than the composite material’s ultimate tensile strength, as well as reported in [13], the [0°/±45°]_6_ lay-up has shown great dynamic response for the composite impeller design. The same conclusion was drawn in [12] where the maximum stress (24 MPa) was found around the blades root of a CFRP impeller, having 180 mm in diameter and a different blade geometry.

The manufacturing technology was selected based on the mechanical and physical properties given to the composite material, by applying pressure, vacuum and temperature simultaneously. A dedicated manufacturing technology was developed, including the metallic moulds design and manufacturing. For assuring the CFRP impeller balancing (high rotational speed component with a very low mass), two metallic rings (304 stainless stee) were integrated on the backside of the impeller disc. The integration of the two metallic rings was found to provide a major advantage during balancing operations of composite rotary structures (in this case a composite centrifugal impeller), as due to the 304 stainless steel higher density (8 kg/m^3^ compared to 1.47 kg/m^3^ of CFRP), a considerable low amount of mass has been removed to properly balance the impeller. According to the reference literature, the proposed design, manufacturing technology and dynamic balancing approach are novel elements for this kind of equipment with complex shapes. As the manufacturing process was taken in two steps, small profile deviations were found at the blade’s tip (±0.386 mm average deviation and a maximum of +0.93 mm), but since these deviations were symmetrical, it was not considered a blocking point for the experimental evaluation of the composite impeller. A total of 45 g were removed from the outer metallic ring during the dynamic balancing operations, indicating that a single metallic ring could be used for balancing a composite compressor impeller. Considering this, a further manufacturing improvement was identified which will lead to a lighter CFRP closed impeller, with at least 177 g, representing the mass for the inner metallic ring. A total of 600% mass reduction was achieved by the composite compressor impeller as compared to the 17-4PH metallic reference impeller, demonstrating the potential mass saving and energy efficiency that could be gained from implementing composite materials to rotary components, as also concluded in [14].

Experimentally measured vibration levels (low and constant values up to rotational speed of 11,353 rot/min) showed that the design, manufacturing and balancing processes of the composite impeller were properly performed. The resonant frequencies were identified at 13.43 Hz 805 rot/min and at 77 Hz with a 0.1 mm/s amplitude at 4400 rot/min, which is lower as compared to [16] where a GFRP mock-up rotor obtained 260 Hz for a 4000 rot/min and 360 Hz for 11,500 rot/min. The identified resonant frequencies are due to electrical motor vibrations/transmission mechanism and were validated by numerical analyses. In conclusion, it can be stated that there is a conformity between the analysis of the frequency response and the vibration monitored during the functional test.

The centrifugal impeller mass reduction corroborated with high mechanical performances represent key aspects in future development of different compressor stages that could benefit from increasing the compression ratio by increasing the tip speed of such impellers. Thus, the proposed new technology responds also to a current trend of substantially increasing the compression of gas turbine compressors in order to reduce fuel consumption and thus reduce CO_2_ emissions.

This paper highlights the feasibility and the advantage of a composite compressor impeller design with application in centrifugal compressors and rotating machine assemblies and sub-assemblies (e.g., gas turbines). Once the manufacturing technology is fully validated, it will be applicable for different sizes of centrifugal impellers that can be widely implemented in aerospace or energy industries.

## 5. Patents

The authors have made a national patent application: Vintila I.S., Mihalache, R., Condruz, M.R., Vilag, V.A., Maier, R., Ansamblu rotativ de compresor centrifugal din materiale compozite polimerice avansate ranforsate cu fibre de carbon, application no. RO 133845 AO. January 2020, which can be accessed here.

## Figures and Tables

**Table 1:** Material properties [26,27].

M49/42%/200T2X2/CHS-3K							
Property	ρ [g/cm^3^]	E [N/mm^2^]	CTE [ppm/°C]	UTS [MPa]	Flexural strength [MPa]	Compression strength [MPa]	ILSS [MPa]
Value	1.47	68,000	2.1 10^−6^	1050	1000	730	60
**Aluminium 2024**							
Property	Density [g/cm^3^]	Thermal conductivity [W/m-C]		Machinability [%]	CTE at 20 °C [ppm/°C]	CTE at 250 °C [ppm/°C]	
Value	2.78	121		70	23.2	24.7	

Where, E—Young Modulus; CTE—Coefficient of thermal expansion; UTS—Ultimate tensile strength; ILSS—Interlaminar shear strength.

**Table 2:** Varying the number of blades and their thickness.

Blades No.	7 Blades			13 Blades			15 Blades			17 Blades		
Thickness [mm]	1×	2×	3×	1×	2×	3×	1×	2×	3×	1×	2×	3×

Where, 1×—reference blade thickness (3 mm), 2×—twice the reference blade thickness (6 mm), 3×—three times the reference blade thickness (9 mm)

**Table 3:** Numerical analysis results for centrifugal impeller varying the blades number and thickness.

Case	7 Blades			13 Blades			15 Blades			17 Blades		
	1×	2×	3×	1×	2×	3×	1×	2×	3×	1×	2×	3×
$\pi_{c}$	1.86	1.96	2.01	2.03	1.94	1.8	2.05	1.91	N/A	2.07	1.82	N/A
*P [kW]*	251	279	355	297	280	285	301	290	N/A	310.4	284	N/A
*η [%]*	78.6	90.1	91.5	90.8	88.8	78.7	91.7	86.4	N/A	91.8	80.7	N/A

Where, π_c_—Compression ratio; P—Power; η—Efficiency

**Table 4:** Scores obtained for calculated geometries.

Case	7 Blades			13 Blades			15 Blades			17 Blades		
	1×	2×	3×	1×	2×	3×	1×	2×	3×	1×	2×	3×
Score [%]	N/A	94.33	103.53	N/A	93.66	88.33	N/A	93.02	N/A	100	89.07	N/A

**Table 5:** Roughness evaluation for the composite blades.

Measurement no.		1	2	3	4	5	6	7	Std. Dev.
Blade	Ra	1.406	1.021	1.318	1.341	1.235	1.331	1.271	0.047
	Rz	22.206	24.336	21.898	22.813	23.315	20.966	24.072	0.457
Disc	Ra	1.306	1.303	1.299	1.322	1.178	1.109	1.227	0.031
	Rz	18.641	24.865	18.069	20.183	17.351	19.449	23.179	1.049

Where, Ra is the roughness average and Rz is the average maximum height of the profile

**Table 6:** Correlation between the maximum relative deviations of the Eigen frequencies determined experimentally and by numerical simulations.

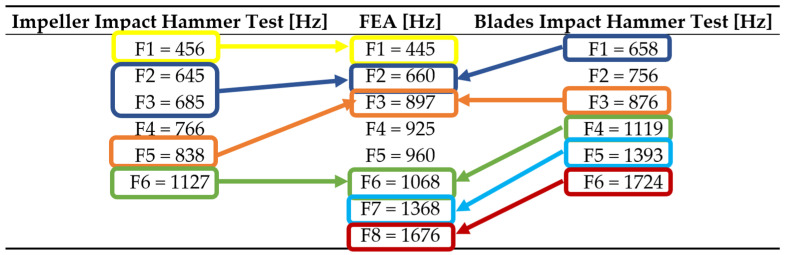

## Data Availability

The data presented in this study are available on request from the corresponding author.

## Nomenclature

*Ø*	Diameter [mm]
*η*	Efficiency [%]
*e*	Compression ratio
*ρ*	Density [g/cm^3^]
1D	One-dimensional
2D	Two-dimensional
3D	Three-dimensional
*b*	Impeller thickness [mm]
*c*	Distance between the mass centre and the furthermost bearing [mm]
*D_max_*	Total displacement [m]
*Ε*	Young Modulus [N/mm^2^]
*e*	Centre of mass deviation from its axis: 0.08
*G*	Balancing class: 2.5
*l*	Distance between bearings [mm]
*M*	Impeller mass with shaft [kg]: 11 kg (impeller mass: 2.271 kg)
*N_e_*	Nominal speed [rot/min]: 17,250 rot/min
*P*	Power [kW]
*S*	Score [%]
*U_c_*	Maximum permissible couple unbalance [g∙mm]
*U_in_*	Initial unbalance [g∙mm]
*U_in,A_*	Initial unbalance in the left (impeller plane) [g∙mm]
*U_per_*	Maximum permissible unbalance [g∙mm]
*U_per,A_*	Permissible unbalance in left (impeller) plane [g∙mm]
*U_per,B_*	Permissible unbalance in the right (skid) plane [g∙mm]
*U_s_*	Maximum permissible static unbalance [g∙mm]
*y+*	Dimensionless thickness

## Appendix A

Calculation of maximum permissible unbalance:

(A1) $$U_{per}=30\times 1000\times \frac{G\times M\;\left[\mathrm{kg}\right]}{\pi \times N_{e}\;\left[\mathrm{rot}/\min\right]}=15.22\;\left[g\cdot \mathrm{mm}\right]$$

Calculation of maximum permissible static unbalance:

(A2) $$U_{s}=\frac{U_{per}}{2}\times \frac{l}{2c}=2.60\;\left[g\cdot \mathrm{mm}\right]$$

(A3) $$U_{c}=\frac{U_{per}}{2}\times \frac{3l}{4b}=19.77\;\left[g\cdot \mathrm{mm}\right]$$

Calculation of permissible unbalance in left plane:

(A4) $$U_{per,A}=\frac{U_{per}\times b}{l}=4.39\;\left[g\cdot \mathrm{mm}\right]$$

(A5) $$U_{per,B}=\frac{U_{per}\times c}{l}=22.26\;\left[g\cdot \mathrm{mm}\right]$$

With the assumption that the impeller’s centre of mass deviation from its axis of rotation is e = 0.08, the initial unbalance is:

(A6) $$U_{in}=e\;\left[\mathrm{mm}\right]\times m\;\left[g\right]=181.68\;\left[g\cdot \mathrm{mm}\right]$$

(A7) $$U_{in,\;\;A}=\frac{U_{in}\times b}{l}=52.44\;\left[g\cdot \mathrm{mm}\right]$$

(A8) $$U_{per,B}=\frac{U_{in}\times c}{l}=265.71\;\left[g\cdot \mathrm{mm}\right]$$

Scoring methodology using impeller compression ratio (2.067), power (310.4 kW) and efficiency:

(A9) $$S=\sum \left(100\cdot \frac{\frac{\pi_{c}}{2.067}+\frac{P}{310.4}+\frac{\eta }{91.80}}{3}\right)$$
